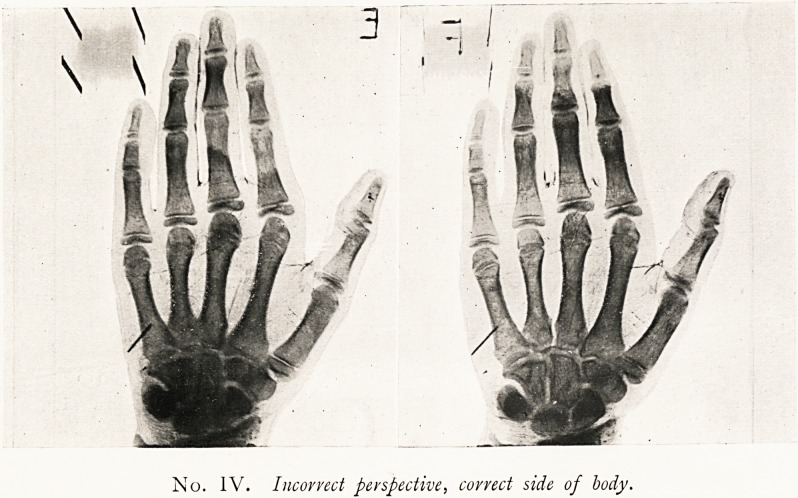# Stereoscopic X-Ray Representation, with an Example

**Published:** 1902-09

**Authors:** William Cotton


### STEREOSCOPIC X-RAY REPRESENTATION, WITH AN EXAMPLE.

BY

WILLIAM COTTON, M.A., M.D., D.P.H.

---

WHATEVER the reasons, the eye does not as a rule appreciate "relief" in the single radiogram. When, therefore, it is necessary to know the position of a foreign body relatively to the bones of the part in the direction of vision, some method of localisation must be resorted to, and undoubtedly the stereoscopic is the most elegant.

A girl aged 12 years, while washing a floor, ran part of a needle (the threaded eye-end, butt foremost as it turned out) into the ulnar aspect of the right hand. The thread was drawn on and snapped short. Generally needles in the soft parts may be left alone; in this case much inconvenience was caused when the patient tried to strike octaves on the piano. There was considerable swelling, and the position of the needle, whether in front of, or at the back of, the metacarpal bones, or otherwise, could not be made out.

The palm of the hand was dusted with bismuth and the hand was twice radiographed, with the palm towards the sensitive film, on separate plates, by a focus tube in two successive positions across the long axis of the hand. These were $2\frac{3}{4}$ inches apart, and the anticathodal centre of emission in each case was 13 inches above the plane of the plate. A wooden cube, one inch in the side, with four metal strips along edges vertical to the plane of the plate, was simultaneously taken (in the same relation to the hand in each case) as a guide to the perspective.

Subsequently the needle was removed easily with the aid of a local anæsthetic, and the patient relieved.

According as we pair the two negatives or prints side by side in the stereoscope, we get *four* distinct and recognisable stereoscopic images. For present purposes the reduced radio-

No. III. *Correct perspective, wrong side of body.*

No. IV. *Incorrect perspective, correct side of body.*

grams in the refracting (or Brewster's instrument) only are considered; but the statements are applicable to the reflecting (or Wheatstone's) as well. These results merit tabulation.

Let the two positions of the focus tube in order be indicated by the letters e and f; then E and F represent the corresponding negatives and Ǝ and ꟻ the corresponding prints:—

RIGHT HAND X-RAYED as described.

| No. | Photographs in order side by side. | Perspective tested by apparent convergence of lines perpendicular to plane of delineation. | Appearance of hand. |
|---|---|---|---|
| I. Negatives. | E F | Correct binocular Perspective. | Right hand through back. |
| II. Negatives. | F E | Distorted and irrational Perspective. | Left hand through palm. |
| III. Prints. | ꟻ Ǝ | Correct binocular Perspective. | Left hand through back. |
| IV. Prints. | Ǝ ꟻ | Distorted and irrational Perspective. | Right hand through palm. |

In all four combinations the *relative* position of the needle in regard to the bones is the same, namely, in front of the fifth metacarpal bone.

Our illustrations give No. III. & IV. Obviously No. I. is the only correct arrangement, though No. IV. would pass muster in a court of law, and (when "relief" in the single print is perceived at all) is generally the accepted perspective. No. II. is the "Conversion of Relief Image" (= exchange of near and far) of No. I.; and No. IV. is the "Conversion of Relief Image" of No. III. The best cure for "Conversion of Relief" in the single photograph is a conversion of belief; namely, that the X-ray print is analogous to the photographic print—whereas it is the X-ray *negative* and the photographic *print* which properly correspond in function. The relations in columns three and four hold good as long as the pairs are inverted together in the same relative order; separate inversion of the single photographs gives at once a different relation, of course. Furthermore, as was to be expected, any pair rendered transparent and looked at from the back (as long as they are in the same relative order) gives the same perspective effect, save only that right becomes left, and left right.

The principles set out in the table hold good with every part of the body; and in ordinary photography in a modified way. Even the notation holds good, so long as we remember that the radiographic *negatives* E and F correspond in perspective with the photographic *prints* E and F.

The illusion known as "Conversion of Relief" in actual vision deserves a fuller consideration elsewhere. It suffices here to point out that the latent images on the two retinæ, being separately inverted, are in the same relation to each other as in a twin stereoscopic camera: that is, in an order tending to produce the illusion in question. One of the puzzles of binocular vision is as to how and where the necessary re-arrangement and re-combination of the separately-inverted images take place.

For the original negatives of the accompanying illustration I am indebted to Mr. Thomas Clark, whose electrical influence machine (as described in this *Journal* for Sept., 1899, and designed by himself) possesses the qualities of definition and steadiness required to produce a pictorial effect; the silhouette of the needle, the surface contour of the palm, and the calcareous skeleton of each bone being clearly represented.

## Figures and Tables

**No. III. f1:**
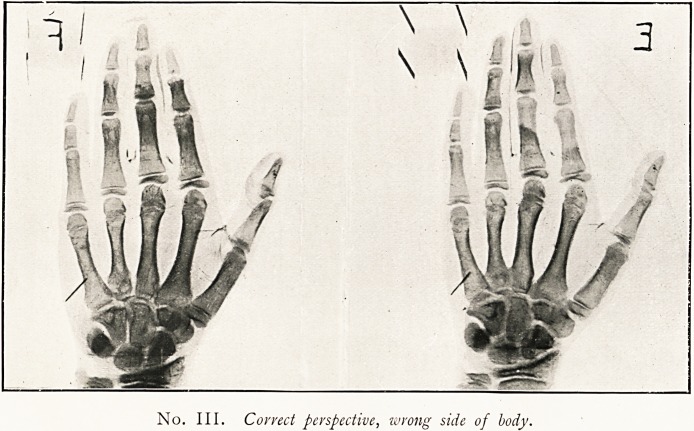


**No. IV. f2:**